# Tetraarsenic hexoxide induces G2/M arrest, apoptosis, and autophagy via PI3K/Akt suppression and p38 MAPK activation in SW620 human colon cancer cells

**DOI:** 10.1371/journal.pone.0174591

**Published:** 2017-03-29

**Authors:** Arulkumar Nagappan, Won Sup Lee, Jeong Won Yun, Jing Nan Lu, Seong-Hwan Chang, Jae-Hoon Jeong, Gon Sup Kim, Jin-Myung Jung, Soon Chan Hong

**Affiliations:** 1 Department of Internal Medicine, Institute of Health Sciences, Gyeongsang National University School of Medicine, 90 Chilam-dong Jinju, Korea; 2 Department of Surgery, Konkuk University School of Medicine, Seoul, Korea; 3 Research Center for Radiotherapy, Korea Institute of Radiological and Medical Sciences, Seoul, Korea; 4 Research Institute of Life Science and College of Veterinary Medicine, Gyeongsang National University, 900 Gajwadong, Jinju, Korea; 5 Department of Neurosurgery, Institute of Health Sciences, Gyeongsang National University School of Medicine, 90 Chilam-dong Jinju, Korea; 6 Department of Surgery, Institute of Health Sciences, Gyeongsang National University School of Medicine, 90 Chilam-dong Jinju, Korea; Duke University School of Medicine, UNITED STATES

## Abstract

Tetraarsenic hexoxide (As_4_O_6)_ has been used in Korean folk medicines for the treatment of cancer, however its anti-cancer mechanisms remain obscured. Here, this study investigated the anti-cancer effect of As_4_O_6_ on SW620 human colon cancer cells. As_4_O_6_ has showed a dose-dependent inhibition of SW620 cells proliferation. As_4_O_6_ significantly increased the sub-G1 and G2/M phase population, and Annexin V-positive cells in a dose-dependent manner. G2/M arrest was concomitant with augment of p21 and reduction in cyclin B1, cell division cycle 2 (cdc 2) expressions. Nuclear condensation, cleaved nuclei and poly (adenosine diphosphate‑ribose) polymerase (PARP) activation were also observed in As_4_O_6_-treated SW620 cells. As_4_O_6_ induced depolarization of mitochondrial membrane potential (MMP, ΔΨm) but not reactive oxygen species (ROS) generation. Further, As_4_O_6_ increased death receptor 5 (DR5), not DR4 and suppressed the B‑cell lymphoma‑2 (Bcl-2) and X-linked inhibitor of apoptosis protein (XIAP) family proteins. As_4_O_6_ increased the formation of AVOs (lysosomes and autophagolysosomes) and promoted the conversion of microtubule-associated protein 1A/1B-light chain 3 (LC3)-I to LC3-II in a dose- and time- dependent manner. Interestingly, a specific phosphoinositide 3-kinase (PI3K)/Akt inhibitor (LY294002) augmented the As_4_O_6_ induced cell death; whereas p38 mitogen-activated protein kinases (p38 MAPK) inhibitor (SB203580) abrogated the cell death. Thus, the present study provides the first evidence that As_4_O_6_ induced G2/M arrest, apoptosis and autophagic cell death through PI3K/Akt and p38 MAPK pathways alteration in SW620 cells.

## Introduction

Colorectal cancer (CRC) is the third most common type of cancer and the second leading cause of cancer related death in the world [1]. CRC represents a major public health problem and the incidence of CRC has recently been increasing especially in Korea [2]. Most of the colorectal cancers belong to the adenocarcinomas accounting with approximately 95% of cases. The 5 years survival rates are very poor for patients, those diagnosed at their advanced stages. Recently survival rates of CRC patients have improved with the help of advanced modality in the cancer research. Despite treatments for CRC including surgery, radiation therapy and/or chemotherapy are generally available, its application still very limited and cause severe side effects [3]. Thus, there is necessity for development of novel therapeutic potential for the CRC prevention and therapy.

Induction of the cell cycle arrest and programmed cell death are the important strategies in the cancer prevention and therapeutics. Apoptosis (type I programmed cell death) and autophagy (type II programmed cell death) are the two major modes of programmed cell death playing an imperative roles in cancer chemoprevention [4, 5]. Both apoptosis and autophagy plays essential roles in development, tissue homeostasis and disease in organisms. The ample evidences indicate that therapeutic agents act through the mechanisms involving cell cycle alteration, apoptosis and autophagic cell death on wide variety of human cancer cell [6–8]. In addition, therapeutic agents may also affects the cell death and survival of multi-signaling pathways within the cells, including Tumor necrosis factor (TNF), TNF-related apoptosis-inducing ligand (TRAIL), PI3K/Akt and mitogen-activated protein kinases (MAPKs) mediated pathways etc [9–12]. Therefore, investigating the mechanism of cell death in colon cancer cells would be helpful to develop novel strategy to treat the colon cancer.

Arsenical compounds have been used in Traditional Chinese Medicines (TCM) for thousands of years to treat a variety of medical diseases, including cancer [13]. Thus, arsenicals have drawn much attention in recent years since its successful clinical application as arsenic trioxide (As_2_O_3_, ATO) for treating acute promyelocytic leukemia (APL) [14]. In addition, As_2_O_3_ exhibited the anti-cancer properties in various cell lines such as gallbladder carcinoma cells [15], mouse embryonic fibroblasts [16], human cervical cancer cells [17], leukemic cell lines [18, 19] and malignant glioma cells [20]. Similarly, tetraarsenic oxide (As_4_O_6,_ TAO) is a natural substance obtained from Sinsuk, and has been used in traditional medicine in Korea for the management of many type of solid cancer. Besides, different from As_2_O_3_, As_4_O_6_ was widely used in Korea in the late 1980’ and 1990’ because it showed some anecdotal cases showing dramatic effects without noticeable side effects. However, only limited studies have exhibited the anti-cancer effect of As_4_O_6_ in human cancer cells, suggesting that the mechanisms of As_4_O_6_-induced cell death are also different from As_2_O_3_ [21, 22]. Our previous study has also demonstrated that As_4_O_6_ has anticancer properties through suppression of NF-κB activity in SW620 cells [23]. However, the anticancer effect and the detailed mechanisms of As_4_O_6_ and its cell regulatory action remain obscured in colorectal cancer.

Here, we have investigated the anti-cancer effect and their molecular mechanism of the As_4_O_6_-induced cell death in SW620 human colorectal cancer cells. Our results provide the novel mechanism which includes As4O6 induced G2/M arrest, apoptosis and autophagic cell death by suppression of PI3K/Akt mechanism and further activating p38 MAPK pathways in SW620 cells.

## Materials and methods

### Cells and reagents

SW620 human colon cancer cells from the American type culture collection (Rockville, MD, USA) were cultured in RPMI 1640 medium (Invitrogen Corp, Carlsbad, CA, USA) supplemented with 10% (v/v) fetal bovine serum (FBS) (GIBCO BRL, Grand Island, NY, USA), 1 mM L-glutamine, 100 U/mL penicillin, and 100 μg/mL streptomycin at 37°C in a humidified atmosphere of 95% air and 5% CO_2_. As_4_O_6_ was obtained from Chonjisan institute (Seoul, Korea). Antibodies against procaspase 3, poly (ADP-ribose) polymerase (PARP), β-catenin, DR4, DR5, Bax, Bcl-2, Bid, cyclin B1, XIAP, p21, AKT 1/2/3 (H-136), phospho-Akt (Ser473), ERK, phospho-ERK (E-4), p53 and Beclin 1 were purchased from Santa Cruz Biotechnology (Santa Cruz, CA, USA). Antibodies against LC3, and Beclin-1, were purchased from PharMingen (San Diego, CA, U.S.A.). Antibodies against phospho-Akt (Thr 308), procaspase 8, procaspase 9, phospho-p38 MAPK, cdc2, JNK, and phospho-JNK were purchased from Cell signaling Technology, Inc. (Beverly, MA, USA). Antibody against β-actin was purchased from Sigma (Beverly, MA). Peroxidase-labeled donkey anti-rabbit and sheep anti-mouse immunoglobulin, and an enhanced chemiluminescence (ECL) kit were purchased from Amersham (Arlington Heights, IL, USA). All other chemicals not specifically cited here were purchased from Sigma Chemical Co. (St. Louis, MO, USA).

### Cell viability assay

The effect of As_4_O_6_ on cell proliferation of SW620 cells was assessed by MTT (3-(4,5-Dimethylthiazol-2-yl)-2,5-Diphenyltetrazolium Bromide) assay [24]. The SW620 cells were seeded onto 12-well plates (10 × 10^4^), and then treated with As_4_O_6_ (0, 0.1, 0.5, 1, 2 and 5 μM) for 24 h and 48 h. MTT powder (0.5 mg/ml) was subsequently added to each well and incubated for 3 h. And then, 500 μl of DMSO was added to dissolve the crystals. The absorption values were determined at 570 nm with an ELISA plate reader. Cell morphology was photographed under light microscopy (magnification, x200).

### Nuclear staining

Morphological changes in cell nuclei was determined by 4,6-diamidino-2-phenylindole (DAPI) staining as described previously [25]. The SW620 cells were seeded in 24-well plates at a density of 5 × 10^4^ cells/mL per well and incubated for 24 h. Cells were treated with As_4_O_6_ at 0, 0.1, 0.5, 1, 2 and 5 μM concentrations, washed the harvested cells with phosphate-buffered saline (PBS) and fixed with 3.7% paraformaldehyde in PBS for 10 min at room temperature. After washed with PBS, the cells were stained with 2.5 μg/ml DAPI solution for 10 min at room temperature. After the cells were washed two times again with PBS, the images of stained nuclei were captured by the NIS-Element AR 3.2 imaging software (Nikon Instruments Inc, NY, USA).

### Propidium iodide (PI) staining for cell cycle analysis and Annexin V-FITC/PI staining for apoptosis by flow cytometry assay

The SW620 cells were plated at a concentration of 2 × 10^5^ cells/well in six-well plates and incubated with As_4_O_6_ at 0, 0.1, 0.5, 1, 2 and 5 μM for 24 h. The cells were collected, washed with cold PBS, and then centrifuged. For PI staining, the pellet was fixed in 75% (v/v) ethanol for 1 h at 4°C. The cells were washed once with PBS and re-suspended in cold PI solution (50 μg/ml) containing RNase A (0.1 mg/ml) in PBS (pH 7.4) for 30 min in the dark. Annexin V double staining was performed according to manufactures instructions. Briefly, 5 μL of the annexin V conjugate was added to each 100 μL of cell suspension for 15 min, and then 400 μL of annexin V-binding buffer was added and mixed gently, keeping the samples on ice. Flow cytometry analyses were performed with beckman coulter cytomics FC 500 (Becton Dickinson, San Jose, CA).

### Measurement of MMP (ΔΨm) and ROS generation

The SW620 cells were seeded onto 6-well plates (2 × 10^5^) and then treated with As_4_O_6_ at 0, 0.1, 0.5, 1, 2 and 5 μM concentrations for 24 h and MMP (ΔΨm) in living cells was measured by flow cytometry with the lipophilic cationic probe JC-1, a ratiometric, dual-emission fluorescent dye [26] There are two excitation wavelengths, 527 nm (green) for the monomer form and 590 nm (red) for the J-aggregate form. The cells were harvested and re-suspended in 500 μL of PBS, incubated with 10 μM JC-1 for 20 min at 37°C. Quantitation of green fluorescent signals reflects the amount of damaged mitochondria. For ROS measurement [27], the cells were incubated with 10 μM 2′,7′-dichlorofluorescein diacetate (DCFDA) at 37°C for 30 min. The cells were then washed with ice-cold PBS and harvested. Fluorescence was determined with beckman coulter cytomics FC 500 (Becton Dickinson, San Jose, CA, USA).

### Western blot analysis

The SW620 cells were seeded onto 6-well plates (2 × 10^5^) and then treated with As_4_O_6_ at 0, 0.1, 0.5, 1, 2 and 5 μM concentrations for 24 h. Total cell lysates were obtained after treated with RIPA buffer (25 mM Tris (pH 7.6), 150 mM NaCl, 1% NP-40, 1% sodium deoxycholate and 0.1% SDS) and protease inhibitors. The protein concentration was determined by Bradford protein assay (Biorad lab, Ricmond, CA, USA). Approximately, 30 μg of lysate was resolved on 10–12% SDS-PAGE, electrotransferred to polyvinylidene difluoride membranes (Millipore, Bedford, MA, USA), and then incubated with specific primary antibodies (1:1000) at 4°C overnight followed by secondary antibody (1:1000) conjugated to peroxidase. Blots were developed with an ECL detection system.

### Inhibitors assay

To identify the role of PI3K/Akt and MAPKs on As4O6-induced apoptosis and autophagy in SW620 cells, where 10 μM SB203580 (Specific p38 MAPK Inhibitor), 10 μM LY294002 (a specific PI3K/Akt inhibitor) were pretreated 1hr before As_4_O_6_ (1 μM) treatment. After incubation with As_4_O_6_ for 48 h, further experiments were conducted.

### Analysis of Acidic Vesicular Organelles (AVOs) with acridine orange staining

The SW620 cells were incubated with As_4_O_6_ at 0, 0.1, 0.5, 1, 2 and 5 μM concentrations for 24 h. Then, the cells were stained with acridine orange dye. Green (510–530 nm) and red (650 nm) fluorescence emission from cells illuminated with blue (488 nm) excitation light was measured by flow cytometry analyses with beckman coulter cytomics FC 500 (Becton Dickinson, San Jose, CA, USA).

### Statistics

Data was expressed as means ± standard deviation (SD). Significant differences were determined using the one-way ANOVA with post-test Neuman-Keuls for more than two group comparison and Student's *t* test for two group comparison. Statistical significance was defined as p<0.05. All experiments were performed in triplicate.

## Results

### Anti-proliferative effect of As_4_O_6_ on SW620 human colon cancer cells

**Fig 1:**
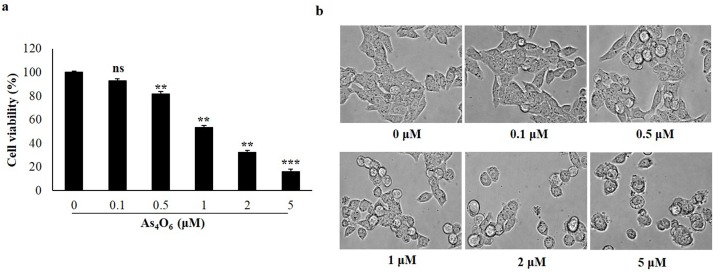
As_4_O_6_ inhibited proliferation of SW620 cells. The cells were seeded at the density of 5x10^4^ cells per ml. (a) The inhibition of cell proliferation was measured by MTT assay. The cells were treated with As_4_O_6_ at 0, 0.1, 0.5, 1, 2 and 5 μM concentrations for 24 h. The antiproliferation of As_4_O_6_ is shown in a dose-dependent manner. (b) Cellular morphology was observed under light microscope (Magnification, X 400). The data are shown as means ± SD of three independent experiments. ‘ns’ represents not significant; ‘*’ represents significance (**p<0.01 and *** p<0.001between the treated and the untreated control group).

To investigate the anti- proliferative activity of As_4_O_6,_ observation was done by 3-(4,5- dimethythiazol- 2-yl)- 2,5-diphenyl tetrazolium bromide (MTT) assay, known to be a colorimetric assay used to determine the reduction of MTT by mitochondrial succinate dehydrogenase in the living cells. In order to assess the cell viability, SW620 cells were incubated with As_4_O_6_ at 0, 0.1, 0.5, 1, 2 and 5 μM concentrations for 24 h and 48 h. The results showed that As_4_O_6_ significantly inhibited the proliferation of SW620 cells in a dose-and time-dependent manner, where the 50% inhibitory concentration (IC50) was estimated approximately at 1μM (Fig 1A & S1 Fig). However, no significant differences were found in cell viability between 24 h and 48 h of As_4_O_6_ treatment. Hence, our further experiments were conducted at 24 h treatment with As_4_O_6_. In order to observe the changes in cellular morphology of As_4_O_6_-treated SW620 cells, examination has been done under light microscopy. The results indicated that the cells with morphological changes such as round shape, cell shrinkage and decrease in cell numbers were observed at 1 μM, 2 μM and 5 μM concentrations of As_4_O_6_ treatment (Fig 1B). These findings suggest that As_4_O_6_ could inhibit the proliferation of SW620 human colon cancer cells.

### As_4_O_6_ induced apoptosis with G2/M cell cycle arrest in SW620 cells

**Fig 2:**
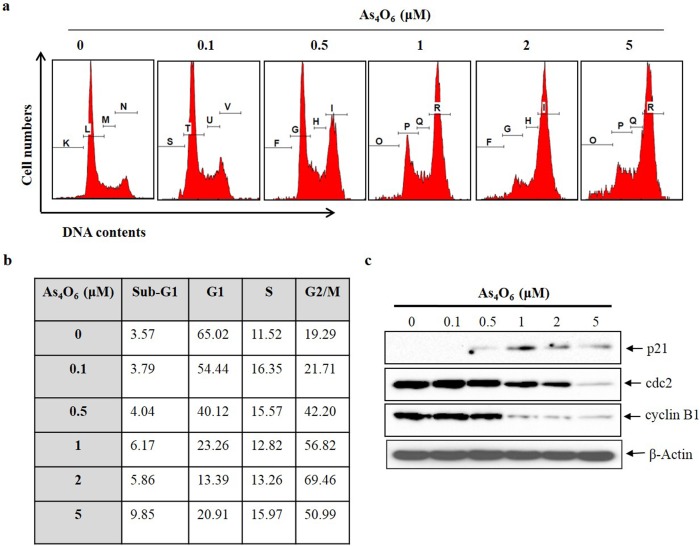
As_4_O_6_ induced G2/M cell cycle arrest of SW620 cells. The cells were seeded at the density of 5x10^4^ cells per ml. (a) To quantify the cell cycle phase distribution, the cells were treated with As_4_O_6_ at 0, 0.1, 0.5, 1, 2 and 5 μM concentrations for 24 h and stained with PI followed by flow cytometry was performed. (b) Quantitative data represents G2/M arrest was induced by As_4_O_6_ on SW620 cells in a dose dependent manner. (c) The cells were treated with As_4_O_6_ at 0, 0.1, 0.5, 1, 2 and 5 μM concentrations for 24 h. Total cell lysates were resolved by SDS-polyacrylamide gels and transferred onto nitrocellulose membranes. The membranes were probed with the p21, cyclin B1, cdc2 antibodies. To confirm equal loading, the blot was stripped of the bound antibody and reprobed with the anti β-actin antibody. The proteins were visualized using an ECL detection system. The data are shown of three independent experiments.

In order to determine, whether the decrease in cell viability of SW620 cells was caused by the induction of apoptotic cell death, flow cytometry was performed. The cell cycle distribution in SW620 cells was examined after treatment with As_4_O_6_ at 0, 0.1, 0.5, 1, 2 and 5 μM for 24 h. The cells with G2/M phase and sub-G1 DNA content were significantly increased in a dose-dependent manner (Fig 2A & 2B). To investigate the further mechanisms responsible for G2/M arrest induced by As_4_O_6_ in SW620 cells_,_ immuno-blotting was performed. The p21, cyclin B1 and cdc2 proteins are crucial in G2/M phase transition process. A complex of cyclin B1 and CDK1 proteins are controlling the G2/M phase, and complex is regulated by cdc25c [28]. The immune-blotting results revealed that As_4_O_6_ increased p21 expression and decreased the expression levels of cyclinB1 and cdc2 proteins in a dose‑dependent manner at 24 h treatment period (Fig 2C). These data suggest that As_4_O_6_ induce G2/M arrest by regulation of p21, cyclin B1 and cdc2 proteins in SW620 cells.

**Fig 3:**
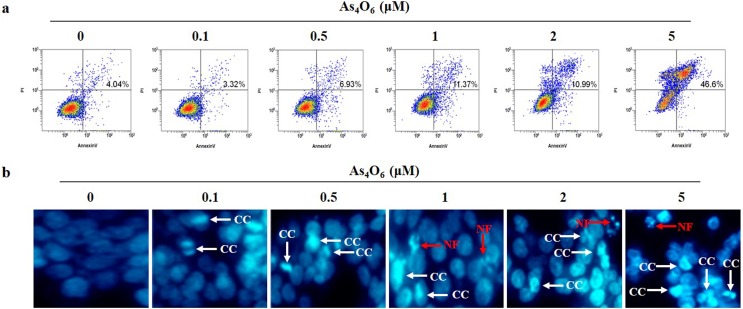
As_4_O_6_-induced apoptosis in SW620 cells. The cells were seeded at the density of 5x10^4^ cells per ml. (a) To quantify the extent of As_4_O_6_-induced apoptosis, the cells were treated with As_4_O_6_ at 0, 0.1, 0.5, 1, 2 and 5 μM concentrations for 24 h and Annexin V was analyzed by flow cytometry. (b) After fixation, the cells were stained with DAPI solution to observe apoptotic body. Stained nuclei were then observed under fluorescent microscope using a blue filter (Magnification, X 400). CC represents chromatin condensation; NF represents nuclear fragmentation. The data are shown of three independent experiments.

Further, to investigate the apoptotic cell death in As_4_O_6_ treated SW620 cells, Annexin V-FITC/PI apoptosis assay was perfomed to confirm that above finding is derived from apoptosis. The obtained results showed that the apoptotic cells were significantly increased in a dose-dependent manner (Fig 3A). As shown in Fig 3B, DAPI staining revealed that normal nuclei of untreated cells had an intact round morphology, whereas the apoptotic nuclei of cells treated with As_4_O_6_ showed chromatin condensation and fragmented nuclei (Fig 3B). These findings suggest that As_4_O_6_ treatment could induce apoptosis in SW620 cells.

### As_4_O_6_ induced the depolarization of mitochondrial membrane potential (MMP, ΔΨm), caspase activation and subsequent cleavage of PARP

**Fig 4:**
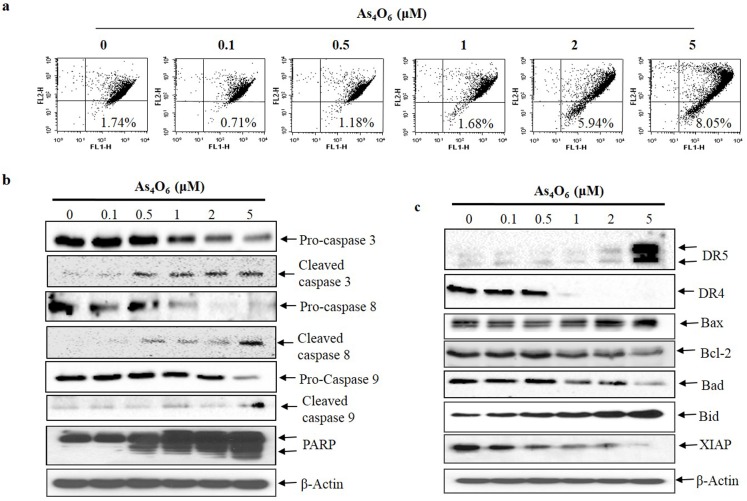
As_4_O_6_ induces MMP (ΔΨm) depolarization, activates of caspases and cleavage of PARP, and DR5 as well as regulation of Bcl-2, IAP family. The cells were incubated at 0, 0.1, 0.5, 1, 2 and 5 μM concentrations of As_4_O_6_ for 24 h. (a) The cells were stained with JC-1 and incubated at 37°C for 30 min. The mean JC-1 fluorescence intensity was assessed by a flow cytometer. As_4_O_6_ induced depolarization of MMP (ΔΨm) at 2 and 5 μM concentrations. (b) Total cell lysates were resolved by SDS-polyacrylamide gels and transferred onto nitrocellulose membranes. The membranes were probed with the anti-caspase-3, anti-caspase-8, anti-caspase-9 and anti-PARP antibodies. (c) The membranes were probed with the antibodies against DR4, DR5, Bcl-2 and IAP family members. To confirm equal loading, the blot was stripped of the bound antibody and reprobed with the anti ß-actin antibody. The proteins were visualized using an ECL detection system. The data are shown of three independent experiments.

In apoptosis induction process, mitochondrial depolarization is important mechanism during mitochondrial mediated pathway[29]. Hence, observation has been taken to measure the changes in mitochondrial membrane potential (ΔΨm) by flow cytometry with JC-1 staining after As_4_O_6_ treatment for 24 h. The results indicated that the depolarization of MMP was induced at 2 and 5 μM of As_4_O_6_ in SW620 cells (Fig 4A). Hence, the finding suggests that As_4_O_6_ -induced apoptosis was associated with mitochondrial pathway in As_4_O_6_ -induced apoptotic cell death. Moreover, ROS generation is also an important mechanism for mitochondria-related apoptosis [29, 30]. Further process was carried out to determine the ROS generation in As_4_O_6_-treated SW620 cells by flow cytometry, where H_2_O_2_ used as a positive control to induce ROS production. The results showed that As_4_O_6_ did not induce ROS production in SW620 cells (S2 Fig), suggesting that As_4_O_6_ induced apoptosis was not due to ROS generation.

To find out whether As_4_O_6_ induced caspase-dependent cell death in SW620 cells, further assessment was done to observe the effects of As_4_O_6_ on caspases protein and their substrate (PARP). Expectedly, western blot analysis revealed that As_4_O_6_ activated the caspase-3, caspase-8, and caspase-9 whereas the cleavages of PARP were clearly observed in a dose- dependent manner (Fig 4B). It has been reported that cleavages of PARP considered as hallmark of apoptosis [31]. These findings revealed that As_4_O_6_ could induce caspase-dependent apoptosis in SW620 cells.

### Effects of As_4_O_6_ on death receptors (DR4 and DR5), Bcl-2 family members and X-linked inhibitor of apoptosis (XIAP)

To investigate whether extrinsic pathways are involved in As_4_O_6_ induced apoptotic cell death, observation was done to examine the expression of TRAIL receptors (DR4, DR5). Western blot analysis revealed that DR5 was up-regulated at 2 and 5 μM of As_4_O_6_ treatment, but not DR4 (Fig 4C). Further, it has been observed that, As_4_O_6_ also suppressed the expression of Bcl-2, and XIAP (anti-apoptotic proteins) whereas an expression of Bax (pro-apoptotic protein) was increased in a dose-dependent manner (Fig 4C). However, As_4_O_6_ did not influence the expression of Bad and Bid.Taken together, these findings suggest that As_4_O_6_ induces apoptosis by up-regulating DR5 which is involved in extrinsic pathway, and that apoptosis is augmented by modulating Bcl-2 and IAP family members which is involved in intrinsic pathway.

### As_4_O_6_ induced autophagic cell death in SW620 cells

**Fig 5:**
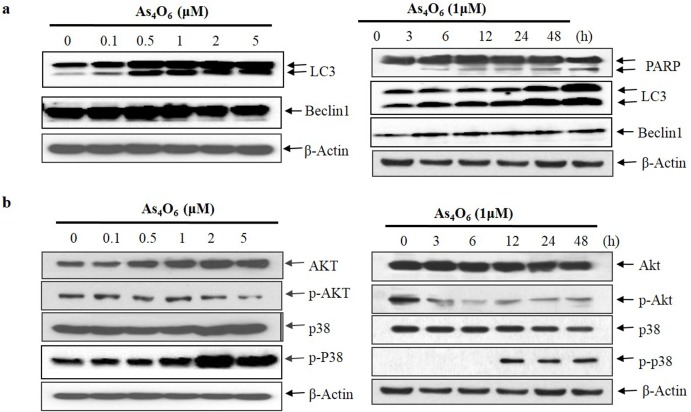
Modulation of autophagy markers, Akt, and p38 MAPK expressions in As_4_O_6_-treated SW620 cells. The cells were treated with As_4_O_6_ at 0, 0.1, 0.5, 1, 2 and 5 μM concentrations for the indicated time intervals. For western blot analysis of (a) Beclin1, LC3, PARP and (b) Akt, and p38 MAPK, equal amounts of cell lysate (30 μg) were resolved by SDS-polyacrylamide gels and transferred onto nitrocellulose membranes. To confirm equal loading, the blot was stripped of the bound antibody and reprobed with the anti ß-actin antibody. The results are from at least three independent experiments that showed similar patterns.

To investigate the autophagic cell death, examination was performed to observe whether As_4_O_6_ treatment induced autophagy in SW620 cells. Further, autophagy markers microtubule-associated protein1 light chain3 (LC3) and Beclin 1 were analyzed by western blot analysis. During autophagy, a cytosolic form of LC3 (LC3-I) is conjugated to phosphatidylethanolamine to form membrane-bound form of LC3 (LC3-II). In this study, two variants of LC3 were detected in western blot, where in the ratio of LC3-II/LC3-I has shown to increase in a dose- and time -dependent manner (Fig 5A; meanwhile, no changes were observed in Beclin 1 in SW620 cells. Interestingly, PARP-1 activation is essential in the autophagy process during the response to chemotherapeutic agent [32]. Hence it has already been observed in Figs 4B & 5A, that As_4_O_6_ induced cleavages of PARP in a dose- and time-dependent manner. In addition, autophagy is characterized by increased formation of AVOs (lysosomes and autophagolysosomes). Hence, formation of AVOs in As_4_O_6_-treated SW620 cells were analyzed by flow cytometry with acridine orange (AO) dye, supporting the obtained result that As4O6-induced the accumulation of AVOs in a dose-dependent manner (S3 Fig). Taken together, the results suggest that As_4_O_6_ induced Beclin-1 independent autophagy by promoting the conversion of LC3-I to LC3-II followed by PARP activation & cleavage in SW620 cells.

### As_4_O_6_ suppressed Akt, and JNK and activated p38, and ERK in SW620 cells

To elucidate the mechanisms for As_4_O_6_–induced cancer cell death, Western blots analysis has been experimented on Akt and MAPKs phosphorylation to determine whether As_4_O_6_ regulates the phosphorylation or dephosphorylation of Akt and MAPKs, which is closely related to cancer cell survival and death [33]. Western blot analysis revealed that As_4_O_6_ remarkably dephosphorylated Akt and JNK in a dose- and time dependent manner (Fig 5B and S4 Fig). Meanwhile, phosphorylation of p-38 MAPK and ERK were significantly increased in a dose- and time dependent manner (Fig 5B and S4 Fig). Moreover, inhibitors of JNK (SP600125) and ERK (PD98059) could not influence the apoptosis induced by As_4_O_6_ in SW620 cells (S4 Fig). Hence, we hypothesized that As_4_O_6_ may induce cell death by suppression of PI3K/Akt and activation of p38 MAPK in SW620 cells.

### Involvement of PI3K/AKT pathway in As_4_O_6_ induced cell death in SW620 cells

**Fig 6:**
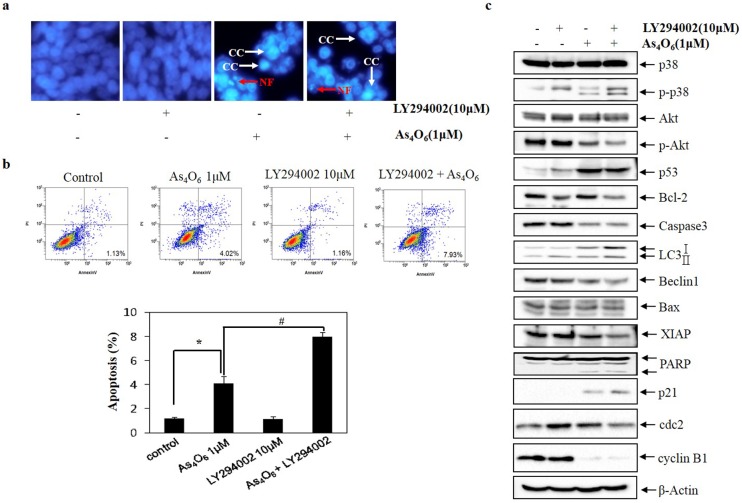
Role of Akt in As4O6 induced cell death in SW620 cells. The cells were treated with LY294002 (10 μM) 30 minute before treatment with As_4_O_6_ (1 μM) for 48 h. (a) To confirm apoptosis, the cells were stained with DAPI solution after fixation. Stained nuclei were then observed under fluorescent microscope using a blue filter (Magnification, X 400). CC represents chromatin condensation; NF represents nuclear fragmentation. (b) Apoptosis was assessed by Annexin V/PI flow cytometry assay. (c) Equal amounts of cell lysate (30 μg) were resolved by SDS-polyacrylamide gels and transferred onto nitrocellulose membranes. To confirm equal loading, the blot was stripped of the bound antibody and reprobed with the anti ß-actin antibody. The data are shown as mean ± SD of three independent experiments. * p<0.05 between the treated and the untreated control group.; # p<0.05 compared between the combination treatment group (LY294002 and As_4_O_6_) and As_4_O_6_ alone treatment group.

Evidence suggests that PI3K/Akt pathway plays pivotal role in regulating cell cycle, apoptosis and autophagy [34, 35]. To assess whether As_4_O_6_ induced cell death was mediated by PI3K/Akt, analysis has been done to observe the cells using flow cytometry after As_4_O_6_ treatment, where the changes in nuclear morphology of As_4_O_6_-treated cells was done by DAPI staining. The DAPI staining revealed that LY294002 (specific PI3K/Akt inhibitor) increased condensed and fragmented nuclei in the As_4_O_6_-treated SW620 cells (Fig 6A). In addition, it has been found that LY294002 augmented the As_4_O_6_-induced cell death (Fig 6B). To confirm this finding at the molecular level, and to determine whether this cell death is associated with PI3K/Akt pathway, western blotting was undertaken. It revealed that the addition of LY294002 to As_4_O_6_ treatment augmented the effects of As_4_O_6_ on cell cycle regulatory proteins: the expression of p21 and cyclin B1, on apoptosis: the expression of XIAP, Bcl-2, PARP cleavage, and on autophagy: LC3 conversion (Fig 6C). Altogether, these findings suggested that PI3K/Akt pathway is involved in As_4_O_6_ induced G2/M arrest and cell death.

### Involvement of p38 MAPK in As_4_O_6_ induced cell death in SW620 cells

**Fig 7:**
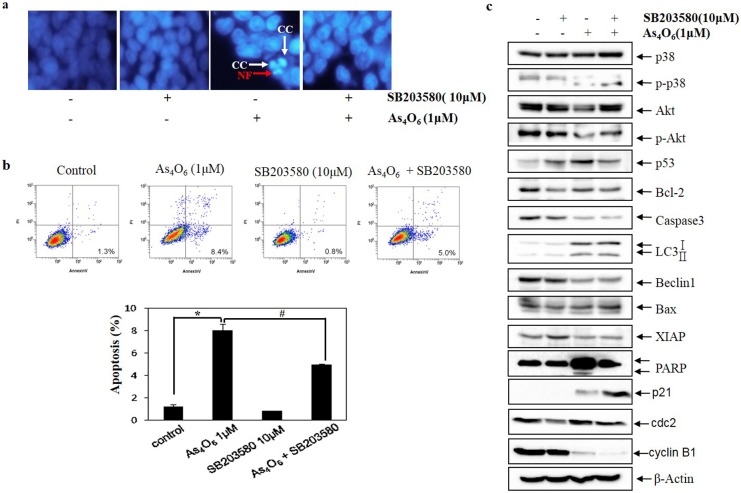
The role of p38 MAPK in As_4_O_6_-induced cell death in SW620 cells. SW620 cells were treated with SB203580 (10 μM) before As_4_O_6_ (1 μM) for 48 h. (a) To confirm apoptosis, the cells were stained with DAPI solution after fixation. Stained nuclei were then observed under fluorescent microscope using a blue filter (Magnification, X 400). CC represents chromatin condensation; NF represents nuclear fragmentation. (b) Apoptosis was assessed by Annexin V/PI flow cytometry assay. (c) The cells were lysed and equal amount of the lysate was separated by SDS-polyacrylamide gels, and then transferred to nitrocellulose membranes. The membranes were probed with the indicated antibodies, and detected by an ECL detection system. To confirm equal loading, the blot was stripped of the bound antibody and reprobed with the anti ß-actin antibody. The data are shown as mean ± SD of three independent experiments. * p<0.05between the As_4_O_6_-treated and the untreated control group; # p<0.05 compared between the combination treatment group (SB203580 and As_4_O_6_) and As_4_O_6_ alone treatment group.

MAPKs signaling pathways are also involved in survival, proliferation, cell cycle, apoptosis and autophagy [36, 37]. ERKs, JNKs and the p38 MAPKs are three major groups of MAPKs. As previously mentioned JNK and ERK could not influence the apoptosis induced by As_4_O_6_ in SW620 cells, the possible role of p38 MAPK in the As_4_O_6_-induced cell death was examined. Hence, the cells were analyzed using flow cytometry after As_4_O_6_ treatment, where the changes in nuclear morphology of As_4_O_6_-treated cells were done by DAPI staining. The DAPI staining revealed that p38 MAPK Inhibitor (SB203580) reduced the frequency of condensed and fragmented nuclei in the As_4_O_6_-treated SW620 cells (Fig 7A). Further, obtained result revealed that p38 MAPK Inhibitor reduced the As_4_O_6_-induced cell death (Fig 7B). To confirm this finding at the molecular level, and to determine whether the Beclin-1-induction is associated with p38 MAPK Inhibitor, western blot examination was carried out for the molecules involved in As_4_O_6_-induced apoptosis and autophagy. It revealed that SB203580 suppressed As_4_O_6_-induced G2/M arrest by restoring the expression of cdc2, and abrogated As_4_O_6_-induced apoptosis and autophagy by blocking the activation PARP and restored the expression of XIAP, and p-Akt (Fig 7C).These findings suggested that the activation of p38 MAPK is also involved in As_4_O_6_ induced G2/M arrest and cell death of SW620 cells.

## Discussion

In our previous study, was demonstrated that anticancer activity of As_4_O_6_ through suppression of NF-κB activity in SW620 cells [23]. The present study was designed to investigate the further mechanisms for the anti-cancer effects of As_4_O_6_, especially on the cell death. The results revealed that As_4_O_6_ induced G2/M arrest, apoptosis and autophagy in SW620 cells. The anti-cancer effects of As_4_O_6_ was associated with PI3K/Akt and p38 MAPK-mediated pathways. To our knowledge, this is the first study report showing multiple anti-cancer mechanisms of As_4_O_6_ in SW620 cells; As_4_O_6_ induced G2/M arrest, apoptosis and autophagy via PI3K/Akt and p38 MAPK-mediated pathways, which is unique from other studies_._

Firstly, As_4_O_6_ exhibited strong anti-proliferative activity on SW620 cells, and induced accumulations of sub‑G1 population (apoptotic cell population) and G2/M phase populations. The G2/M phase progression is controlled by cyclin B1 and CDK1 complex, which is regulated even by cdc25c. Here, in this study obtained immuno-blotting results indicated that As_4_O_6_ significantly increased p21 and decreased cyclin B1, and cdc 2 proteins expression in SW620 cells. Previous studies have also demonstrated that the anti-proliferative effects of arsenic compounds were linked to a G2/M phase cell cycle arrest in various cancer cells[38, 39]. Hence these results indicate that As_4_O_6_ induced G2/M arrest might be associated with down-regulation of cyclin B1 and CDK1 complex and up-regulation of p21.

In addition, there is an association with accumulation of G2/M phase population and apoptosis [40]. Hence, As_4_O_6_‑induced apoptosis was confirmed by FITC‑Annexin V and PI double staining, nuclear condensation, cleaved nuclei and PARP activation. These findings suggest that As_4_O_6_ could induce apoptosis in SW620 cells. To elucidate the molecular mechanism of anti-cancer effect of As_4_O_6_, immune-blotting was performed. Apoptosis can be executed through either extrinsic pathway and/or intrinsic pathway [41]. In this study, As_4_O_6_ significantly activated the caspase -3, -8 and -9 and induced PARP cleavage has been observed in SW620 cells (Fig 4A). The pattern of cleavages in PARP (89 kDa), can be distinguished from necrosis or other type of cell death, which indicated that As_4_O_6_-induced cell death is caspase-dependent apoptosis [42]. The pathway is involved in mitochondrial outer membrane permeabilization cofers a critical event in apoptosis [43]. The up-regulated of DR5 and activation of caspase 8 by As_4_O_6_ suggests that As_4_O_6_ induced apoptosis through extrinsic pathway. Apart from death receptors, mitochondrial membrane potential is also important in inducing apoptosis. Further, our results showed that As_4_O_6_ induced depolarization of mitochondrial membrane potential (MMP), but not ROS generation. Taken together, As_4_O_6_-induced apoptosis in SW620 cells might be contributed through extrinsic pathway via DR5 up-regulation as well as mitochondrial mediated intrinsic pathways. This apoptosis was augmented by the modulation of Bcl-2 and IAP family members, which support our results that As_4_O_6_ induced apoptosis might attributed through multiple mechanisms.

The findings of this study also suggested that the As_4_O_6_ induced autophagy or type II programmed cell death could be another mechanism for As_4_O_6_ induced cell death. In this study, two variants of LC3 were detected in western blot, where in the ratio of LC3-II/LC3-I increased in a dose- and time-dependent manner (Fig 5A). Although this finding appears similar to As_2_O_3_ in colon cancer cell death [19, 44] it is different from As_2_O_3-_induced cell deaths, which is Beclin-1-independent autophagic cell death. However, it may not be a unique finding of As_4_O_6_-induced autophagy because it recently has been reported that arsenic trioxide also induces a Beclin-1-independent autophagic cell death in ovarian cancer cells [45]. In addition PARP-1 activation is also an essential process in the autophagy during the response to chemotherapeutic agent [32]. Our results also showed similar patterns that the conversion of LC3-I to LC3-II and PARP activation & cleavage. These results revealed that autophagy might contribute to the anti-cancer activity of As_4_O_6_.

Regarding up-stream signaling, it was demonstrated that As_4_O_6_-induced apoptosis is closely related to activation of p38 MAPK and suppression of Akt activities. MAPKs composed of three major groups (ERKs, JNKs and the p38 MAPKs) that are mainly involved in cell survival, proliferation and apoptosis [36, 37]. Hence, we examined the phosphorylation status of MAPKs by western blotting to elucidate the molecular mechanisms that are involved in As_4_O_6_-induced apoptosis in SW620 cells. The present study showed that As_4_O_6_ dephosphorylated JNK, and phosphorylated p38 and ERK, but JNK and ERK signaling pathways was not involved in As_4_O_6_-induced cell death. The cell death was blocked by p38 MAPK inhibitor; suggesting p38 MAPK was associated with As_4_O_6_-induced cell death (Fig 6). Different from previous study, we found that the activation of p38 MAPK was associated with As_4_O_6_-induced cell death of SW620 cells. In general, the activation of p38 MAPK is associated cell cycle arrest and apoptosis at early time of treatments. Furthermore, our study demonstrated that As_4_O_6_ dephosphorylated Akt, which has been reported in regulating cell proliferation and apoptosis, and that a specific PI3K/Akt inhibitor augmented As_4_O_6_-induced cell death in SW620 cells (Fig 7). These findings suggest that PI3KAKT pathway also involved in As_4_O_6_-induced cell death of SW620 cells. Taken together, our findings support that p38 MAPK and PI3K/Akt pathways might be attributed to the As_4_O_6_-induced cell death of SW620 cells.

The limitation of this study is that some scientists believe that there is no functional difference between As_4_O_6_ and As_2_O_3_ in dissolved status. In addition, in human body, 3+ form changes into 5+ form and even these two are changing from one into another and they also get methylated in human body. Furthermore, this oxidation of As3+ into As5+ was variable depending on growth media or expose to light conditions. Here, we did not clearly determine the oxidation status of As_4_O_6_. Here, we just focus on the mechanisms of the anti-cancer effects of As_4_O_6_ to explain the case showing some clinical improvement; this study helps to understand the clinical cases showing long-term stable disease with central necrosis. It could be explained by p38 MAPK-associated dormant status with apoptosis and autophagy that was related to Akt/PI3K.

## Conclusions

In the present study, we have demonstrated that the anti-cancer mechanism for As_4_O_6_-induced G2/M arrest, apoptosis and autophagy in SW620 cells. Furthermore, As_4_O_6_ suppressed the PI3K/Akt and activated the p38 MAPK. These activities might play a critical role in As_4_O_6_-induced cell death of SW620 cells. Lastly, this study provides the evidence that As_4_O_6_ has an anti-cancer effects; it might be useful for the understanding the clinical cases showing long-term stable disease with central necrosis.

## Supporting information

S1 Fig As_4_O_6_ cells time-dependently inhibited proliferation of SW620 cells. The cells were seeded at the density of 5x10^4^ cells per ml. The inhibition of cell proliferation was measured by MTT assay. The cells were treated with As_4_O_6_ at 0, 0.1, 0.5, 1, 2 and 5 μM concentrations for 24 h and 48 h. The anti-proliferation of As_4_O_6_ is shown in a dose- and time- dependent manner. The data are shown as means ± SD of three independent experiments. ‘ns’ represents not significant; ‘*’ represents significance (**p<0.01 and *** p<0.001between the treated and the untreated control group).
(TIF)

S2 Fig As_4_O_6_ did not induce ROS generation in SW620 cells. For the assessment of ROS level, the cells were incubated with 10 μM DCF-DA for 30 min after As_4_O_6_ (2 μM) treatment. H_2_O_2_ (2Mm) was used as positive control. The fluorescence intensity was assessed by a flow cytometer.
(TIF)

S3 Fig Effect of As_4_O_6_ on the autophagy in SW620 cells. The cells were treated with As_4_O_6_ at 0, 0.1, 0.5, 1, 2 and 5 μM concentrations for 24 h. After incubation, the cells were stained with 5 μg/mL acridine orange for 17 min and collected in phenol red-free growth medium. Green (510–530 nm) and red (650 nm) fluorescence emission illuminated with blue (488 nm) excitation light was measured with a flow cytometer. As_4_O_6_ induced dose-dependent AVO formation in SW620 cells.
(TIF)

S4 Fig Role of ERK and JNK in As_4_O_6_ induced cell death in SW620 cells. The cells were treated with ERK inhibitor, PD98059 (20 μM) and JNK inhibitor, SP600125 (10 μM) 30 minute before treatment with As_4_O_6_ (1 μM) for 48 h. (a) For western blot analysis, equal amounts of cell lysate (30 μg) were resolved by SDS-polyacrylamide gels and transferred onto nitrocellulose membranes. To confirm equal loading, the blot was stripped of the bound antibody and reprobed with the anti ß-actin antibody. The data are shown as mean ± SD of three independent experiments. ‘ns’ represents not significant; ‘*’ represents significance (**p<0.01 between the As_4_O_6_ treated and the untreated control group.
(TIF)
