# Supplementary figures and images for: Tetraarsenic hexoxide induces G2/M arrest, apoptosis, and autophagy via PI3K/Akt suppression and p38 MAPK activation in SW620 human colon cancer cells
Source: PLoS One. 2017 Mar 29;12(3):e0174591. doi: 10.1371/journal.pone.0174591 (PMC5371332; doi:10.1371/journal.pone.0174591)

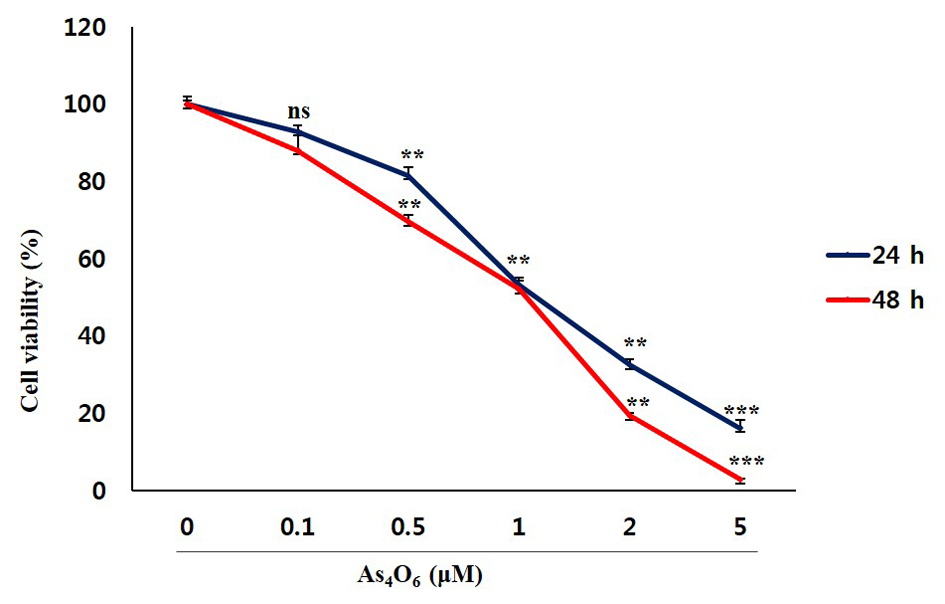

Supplement: S1 Fig — The cells were seeded at the density of 5x10^4^ cells per ml. The inhibition of cell proliferation was measured by MTT assay. The cells were treated with As4O6 at 0, 0.1, 0.5, 1, 2 and 5 μM concentrations for 24 h and 48 h. The anti-proliferation of As4O6 is shown in a dose- and time- dependent manner. The data are shown as means ± SD of three independent experiments. ‘ns’ represents not significant; ‘*’ represents significance (**p<0.01 and *** p<0.001between the treated and the untreated control group). (TIF) [file pone.0174591.s001.tif]

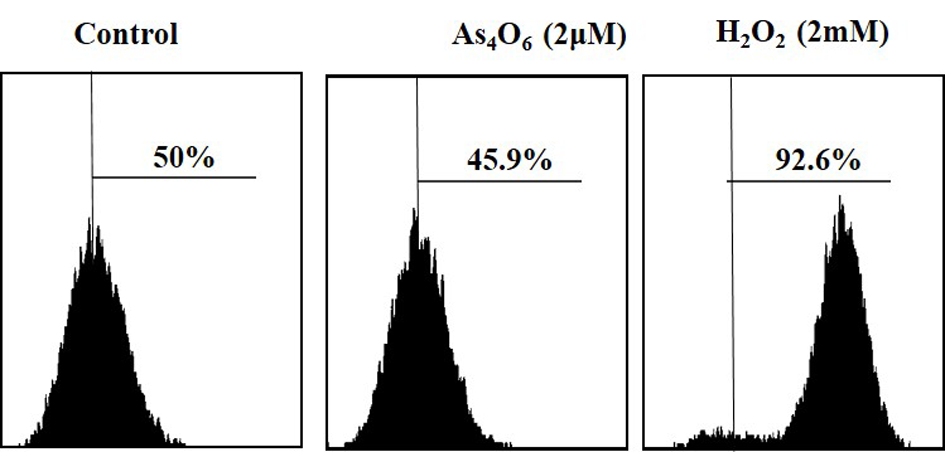

Supplement: S2 Fig — For the assessment of ROS level, the cells were incubated with 10 μM DCF-DA for 30 min after As4O6 (2 μM) treatment. H2O2 (2Mm) was used as positive control. The fluorescence intensity was assessed by a flow cytometer. (TIF) [file pone.0174591.s002.tif]

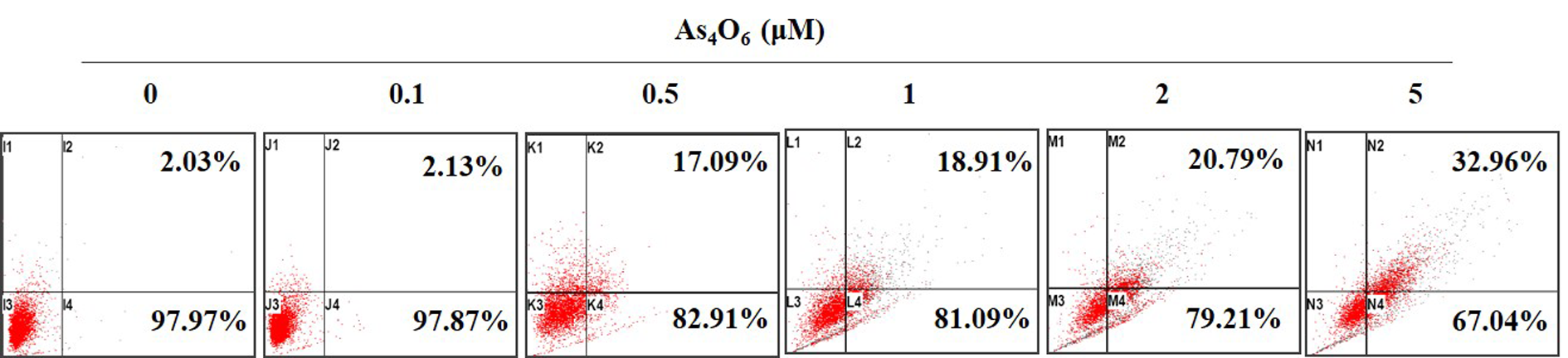

Supplement: S3 Fig — The cells were treated with As4O6 at 0, 0.1, 0.5, 1, 2 and 5 μM concentrations for 24 h. After incubation, the cells were stained with 5 μg/mL acridine orange for 17 min and collected in phenol red-free growth medium. Green (510–530 nm) and red (650 nm) fluorescence emission illuminated with blue (488 nm) excitation light was measured with a flow cytometer. As4O6 induced dose-dependent AVO formation in SW620 cells. (TIF) [file pone.0174591.s003.tif]

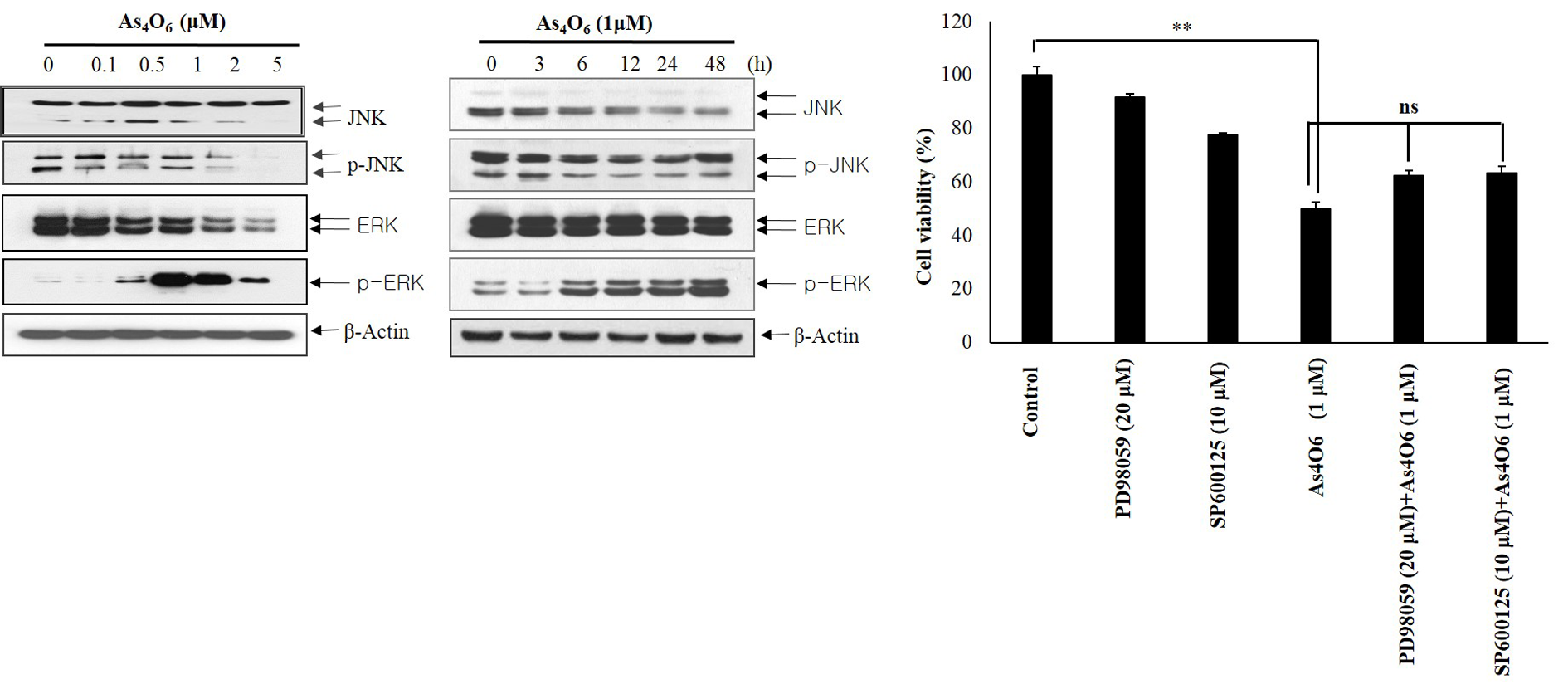

Supplement: S4 Fig — The cells were treated with ERK inhibitor, PD98059 (20 μM) and JNK inhibitor, SP600125 (10 μM) 30 minute before treatment with As4O6 (1 μM) for 48 h. (a) For western blot analysis, equal amounts of cell lysate (30 μg) were resolved by SDS-polyacrylamide gels and transferred onto nitrocellulose membranes. To confirm equal loading, the blot was stripped of the bound antibody and reprobed with the anti ß-actin antibody. The data are shown as mean ± SD of three independent experiments. ‘ns’ represents not significant; ‘*’ represents significance (**p<0.01 between the As4O6 treated and the untreated control group. (TIF) [file pone.0174591.s004.tif]
